# Reviving medical tourism in Pakistan - A narrative review

**DOI:** 10.12669/pjms.40.11.8673

**Published:** 2024-12

**Authors:** Nazia Mumtaz, Ghulam Saqulain

**Affiliations:** 1 Nazia Mumtaz, PhD (Rehabilitation Sciences) Head of Department & Professor, Department of Speech Language Pathology, Faculty of Allied Health Sciences, Riphah International University, Lahore, Pakistan; 2 Ghulam Saqulain, FCPS (Otorhinolaryngology) Head of Department & Professor, Department of Otorhinolaryngology, Capital Hospital PGMI, Islamabad, Pakistan

**Keywords:** Clinical services, Healthcare, Joint Commission International, Medical Tourism, Rehabilitation, Speech therapy

## Abstract

Medical and health care traverse geographical boundaries in the form of “Medical Tourism” with patients travelling from low and middle-income countries to developed nations and vice versa as well. Affordable medical care is also attracting patients from developed nations to countries like India, Thailand, UAE and others with international accreditation playing a key role. This also yields economic benefits for the recipient countries. Pakistan unfortunately lags behind in acquiring “destination of choice” status for medical tourism which undisputedly can benefit its constrained economy. There being a dearth of literature current narrative review was conducted to highlight the integrity and benefits of medical tourism. For this, literature search was conducted using google, Bing, google scholar, PubMed, web of science to search engines and data bases. One hundred eighty articles, reports & publications were downloaded of which 25 non-English & duplicates, and 75 irrelevant records were excluded. Hence, 34 references of relevant English language articles, publications, reports and online resources were utilized for the review.

## INTRODUCTION

The last two decades has seen the concept of tourism for medical purposes evolve. Medical tourism is defined as the practice of travelling across borders for medical treatment.^1^ In simple parlance in “Medical Tourism” patient travels off shore to another country to obtain medical care. “Medical Tourists” is a term used by medical centers in recipient countries for those individuals from one country to another country. This is interestingly not only limited to patients going from low and middle income countries to centres of excellence in developed nations, but availability of cheaper medical care also attracting patients from developed nations to countries such as India, Thailand, United Arab Emirates for medical and other health care related needs which is not just limited to dentistry, fertility issues etc. Costs of medical care abroad are sometimes blurred between the healthcare systems of recipient and originating country when patients from developed countries like United Kingdom obtain treatment abroad and return to their own country for treating complications which instead causes an inequity in the health systems of the originating country.^2^

The essential requirement to be awarded the status of “destination of choice” for medical tourism is for a healthcare institution, clinic or laboratory to acquire international accreditation thus inspiring trust and assurance for patients intending to travel for such treatment. This is in fact beneficial for the institutes, clinics, laboratories since they also grow in terms of human resource, finances and technical upgradation. In most countries offering medical tourism, this has resulted from the private sector being at the forefront though the involvement of governments is advantageous and has wide scope.^3^ Medical tourism earns huge revenues for states facilitating it as well as there are associated economic advantages for hospitality and travel industry too.^4^

It was highlighted in a 2015 Medical Tourism Association Medical Tourism patient survey that healthcare costs on an average remains in the range of USD $3600 to $7600 on medical healthcare on a single medical tourism sojourn abroad. Health care costs are affordable in Thailand for the residents of USA as spinal fusion costs fluctuate from USD $62000 in USA to as low USD $7000.^5^ The reduced costs and waiting time and technology are resultantly changing the dynamics of this growing industry.^1^ It is high time for Pakistan, being a developing country, to strive for its share in medical tourism to benefit its constrained economy. Also, the research gap persists in Health Tourism research as identified by Crooks VA et al.^6^ Hence, the current review was conducted to highlight the integrities and benefits of medical tourism. The study is important since it may initiate discussions, mobilize public opinion and spur policy decision making to help develop Pakistan as a Medical Tourist Hub benefiting it by improving its health care system and economy.

## METHOD

This narrative review was conducted with a literature search using google, Bing, google scholar, PubMed, web of science search engines and data bases utilizing keywords like medical, tourism, travel, healthcare, and combination of terms. For this 180 records were downloaded of which 25 duplicate and non-English leaving behind 155 pertinent English articles, material, reports, online resources and publications which were skimmed to identify 80 relevant, English language, full text documents out of which 33(18.33%) were reviewed in detail utilized for the current narrative review. (Figure). Keeping in view the importance of the topic, no limitation of time period was placed.

**Fig.1:**
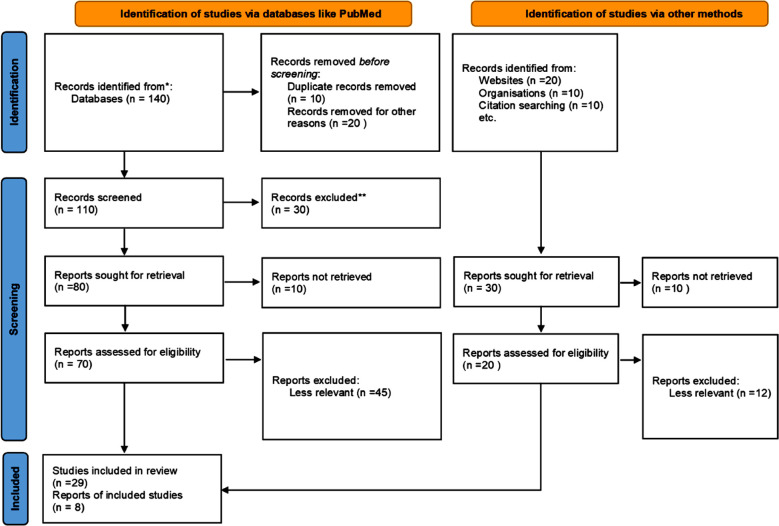
PRISMA flow diagram.

## DISCUSSION

To improve the economy and health care system in the current world, Pakistan needs to transform to a Medical Tourist Hub. For this current study reviewed the literature to highlight the integrity and benefits of medical tourism. This may help realize and reenact medical tourism in Pakistan. For this, we discuss below Medical Tourism in Pakistan & Asian States; Accreditation including i) International accreditation, ii) E-health accreditation, iii) Profits and JCI accreditation; Extent and scope of medical tourism; Lack of evidence around medical tourism; Risks of medical tourism; JCI supports medical tourism; & Rehabilitation and Brain Drain:

### Medical tourism: Pakistan & Asian states:

Pakistan lags behind as regards medical tourism which is restricted to Pakistanis living in England opting for treatment in Pakistan due to long waiting time of the UK National Health Services and also due to the fact that they still have relatives in Pakistan, where they avail cheaper treatment which also includes dental and cosmetic procedures. Though Pakistan’s reputation is improving in medical tourism yet lags far behind in becoming a regional medical tourism hub. The reasons behind this might be that majority of health care institutions, medical centers and laboratories lack international accreditation, making patients uncomfortable since they lack assurance of provision of care conforming to international regulatory parameters.^7^

International accreditation achieved by regional South Asian states and neighboring India, Thailand and Malaysia have resulted in high returns on investments as regards accreditation and are thus booming with medical tourism.^8^ On the other hand the Korean government realized that medical tourism could be a significant industry and evolved medical, legal, and policy strategies with the goal of ultimately soliciting 700,000 foreign patients by the year 2027. In this backdrop integrated two projects were converted into a Wellness and Medical Tourism Convergence Cluster strategically run at three major cities. South Korea lagged behind many Asian countries and realizing this it achieved excellence in the field of cancer surgery and cancer treatment with its medical prowess surpassing countries such as England, Spain, Italy, the Netherlands, Germany, France, and England, United States and Canada. South Korea’s extremely well developed health care systems based on national health insurance may have increased its overall competitiveness both in price and in cancer treatment strengthened by issuance of visas for foreigners, hospital certification system and its medical tourism convergence cluster project.^9^

The scope of medical tourism can cover surgical procedures as well as rehabilitation of stroke patients along with the treatment and management of speech, hearing, communication and swallowing disorders, the availability of which is widespread in Pakistan.

### Accreditation:

### International accreditation:

A number of international accreditation organizations including Joint Commission International (JCI) of USA are recognized and contacted for accreditation in South Asia, Far and Middle East.^4^ The JCI of United States accreditation demands compliance to standards and bench marks with detailed inspection be it a hospital, laboratory, research center and/or other clinical care programs like dental implant clinic, mental care center, ENT center, diabetes units, stroke center, communicable diseases center or palliative care or an academic medical center.^10^

Though owners of hospital setups consider accreditation as a tool for marketing, the health professionals look at these programs as demanding and more bureaucratic with concerns regarding cost of accreditation.^11^ Still, Joint Commission International has accredited more than one thousand programs in 70 plus nations in Asia, Middle East, Europe, Africa, and Latin America.^12^ For accreditation, the applicant organization has the option to apply to JCI for accreditation of entire hospital or specify a specific unit of hospital for accreditation. This follows JCI inspection which includes evaluation of quality of clinical services, safety of patient, assessment of competence of clinical and administrative manpower, assessment of quality of equipment and scrutiny of policies and protocols and procedures being employed. Further to maintain accreditation institutions may face snap inspections. The hospitals may apply for re-accreditation and comply and conform to latest criteria.^12^ There are challenges to JCI accreditation however accreditation positively impacts culture of patient safety and accredited hospitals demonstrate improved team working, communication as well as involvement of patients in hospital care.^13^

### E-Health Accreditation:

E-Health is a neglected multidisciplinary field in Pakistan. Pakistan has a population of around 230 million justifying E-health as a cost effect service which can support healthcare. During the Covid-19 pandemic E-health usage was developed to some extent especially for the rehabilitation services, however its use almost relapsed back to pre-pandemic period with dearth of access to such services in the far-flung areas. There are ethical concerns in use of such E-health data and the World Health Organization and United Nations Educational Scientific and Cultural Organization have given ethical guidelines for provision of health information which has been made digitally available for the public. The scope and coverage of E health is useful for complementing rehabilitative services like speech therapy to patients in far flung areas not easily accessible and not having enough density of population to justify physical presence of such clinical services.^14^ The international medical tourists balance low cost of medical treatment with high-quality medical service destination wise – Digitization and international tele medicine as a form of Medical Tourism is Recommended

### Profits and JCI accreditation:

Medical Tourism has positive financial impact on recipient economies and growth.^15^ Medical tourism worldwide is estimated to generate revenues in the range of 60-70 billion US dollars and in spite of having reliable and competent medical professionals and medical, rehabilitative and healthcare services Pakistan is not grabbing any significant proportion of the revenue with the bulk going to India, Malaysia, Thailand, Costa Rica, Turkey, Mexico etc.^16^ The Indian sector alone is worth one billion US dollar estimated by 2012.^17^

The need brought forward by medical tourism is a stimulant for improving the quality of healthcare measured against common health-quality metrics worldwide considering factors of accessibility and risks of acquiring post-operative infection. Pakistan can have a slice of the pie and enter the sector of medical tourism but it would need to have more of its institutes as JCI accredited. A medical tourist is not alone and accompanied usually by his family members and in case of minor procedures social tourism is an additional agenda.^18^

### Extent and scope of medical tourism:

The economic aspect of medical tourism cannot be ignored. More revenues require more research in the medical and public health domain. Advancement in medical technology and machines leads to faster and accurate diagnosis and improved treatment. The increase in air travel over the last few decades has increased the patient and healthcare professionals flow across international borders with changing patterns of medical tourism, however this can also result in risks for patients.^19^,^20^ Medical tourism patterns are changing with improving technologies and skills in different countries with a number of Asian states becoming dominant and others entering the market.^21^

According to Lunt et al. & Carrera et al, in Medical Tourism the patient as a medical trade consumer conscientiously and by his will opts to travel for medical treatment across borders. Most popular treatments availed include cosmetic and dental surgical procedures and treatment for fertility as well as better quality transplant and elective surgical option with cost being an important deciding factor. On account of standardization of procedures and JCI accreditation patients from wealthier and advanced nations prefer obtaining treatment in less developed countries at significantly reduced expenditures.^22^

### Lack of evidence around medical tourism:

Despite a number of media hypes, evidence on the role and impact of medical tourism for which Organization for Economic Cooperation and Development (OECD) countries work together for the promotion of sustainable economic growth and development is not abundantly available. The evidence available to date with OECD academic institutes suggest that medical tourism includes a range of attendant risks and opportunities for patients and highlights the degree of involvement of a mushrooming industry of intermediaries and ancillary services.^23^ Caution needs to be exercised in Medical Tourism treatment processes since quality of care and risks involved may have implications for this trade and services in the country of origin as well. This requires monitoring and research to determine efficacy of such travel for medical needs, regulatory issues and standardization of medical facilities in host states before considering and promoting medical tourism to such countries.^24^ Authentic data on the scope and success of such tourism is lacking in reliability and epidemiological data shows statistics of 60-70 billion US dollars generated due to medical tourism in the year 2015 alone.^25^

### Risks of medical tourism:

The basic compulsion of medical tourists is low costs in recipient countries like India to get management for a condition or simply get a procedure in recipient country at low cost. These procedures may at times be risky, may be new innovations, or even procedures like traditional Indian healing or yoga for a condition requiring standard medical regimen.^26^,^27^

To obtain cheap and convenient treatment medical tourists at times overlook the risks involved in acquisition of treatment like infections as the surgical interventions they may receive from recipient countries may vary and tourists may be at risk with varying immunity level against infections of wounds, blood born infections, donor related infections and diseases like hepatitis and AIDs. Similarly, the elements of licensure maintenance, credentialing as well as accreditation may have been marked at variance compared to that need in the United States, even if it doesn’t negatively impact patient care. Also, there is risk of immediate travel following invasive surgical procedures with complications like intravascular coagulation and thrombosis which can take place hence post op follow up may be difficult in another country.^28^

From an academic and industry perspective medical tourism is a field which has not been extensively researched. Medical tourism has to be accompanied with leisurely and comfortable tourism to attract international patients. The Asian financial crisis of 2008 drastically reduced the paying capacity of Asians to obtain private healthcare compelling private medical institutions to shift their marketing practices towards international patients in order to generate revenue. The respective governments also supported these actions on the premise of generating valuable foreign exchange. Presently 28 countries are competing worldwide or perhaps on a regional grid for the medical tourism business.^29^ Developing countries which are engaged in facilitating medical tourism present a combination of regulation and policies to support the industry. However, there is an ugly side as well including illegal services like organ sales and services which are deemed illegal in the patient’s own country but legal in the recipient country like stem cell therapies etc.^30^

### JCI supports medical tourism:

Joint Commission International has been instrumental in global improvement in quality and safety in health care for the last few decades and is striving for improving such services through education, advisory service, provision of solutions online and providing accreditation and certification. The JCI accreditation provides comfort and assures the tourist that their destination country like India, Thailand, Turkey, United Arab Emirates etc., have the necessary level of accreditation which is advantageous for the tourist. Hence, all such hospitals in destination countries try to obtain JCI accreditation since their aim is to gain revenues by attracting tourists and retaining highly trained manpower.^31^,^32^

In Pakistan four hospitals are JCI accredited namely Agha Khan University Hospital Karachi, Shifa International Hospitals Ltd. Islamabad, Shaukat Khanum Memorial Cancer Hospital and Research Centre Lahore and Shaukat Khanum Memorial Cancer Hospital and Research Centre Peshawar. Pakistan, the sixth populous country in the world, has no public sector hospital or medical center or research center with JCI recognition.^31^ Worldwide and especially in tourism hubs the JCI hospitals and institutes and centers include Indonesia 30, Malaysia 16, Singapore 8, Japan 30, Turkey 46, Pakistan 7, Saudi Arabia 111, Qatar 22, Oman 7, UAE 212, China 46 and Bangladesh with two. India’s health policy makers have in a concerted manner supported in rapidly acquiring a status of “Preferred Healthcare Destination” and healthcare centers, laboratories and hospitals are attempting to acquire the ‘Gold Seal of Approval’ from JCI.^31^

### Rehabilitation and brain drain:

A study by Kirigia JM et al. in 2006 revealed that the cost incurred to produce a medical graduate from primary onwards is of an amount of 65,997 US dollars and if the doctor leaves the country approximately 517,931 US dollars of return on the initial investment goes down the drain. Similarly, for any nurse that emigrates to a developed country the return on the initial investment is in the range of 338,868 dollars which is indirectly financed by every citizen of the developing country. Such estimates of financial losses do not capture invisible costs (interests on bank loans) of medical education.^33^

As developed countries continue to plunder scarce medical and health human resource of countries such as Pakistan and carry out poaching at will the mindset that has developed and become entrenched in the minds of leading healthcare experts is that centers of medical, health and academic excellence cannot be established in Pakistan. The same experts extol the virtues of conducting research on the huge populace and unethically sharing with academia in the developed nations. National pride has taken a backseat it seems amongst medical professionals and they are content to play second fiddle internationally being shamefaced of the academic institutes of which they are graduates.

Pakistan, though a developing country, has an increasing pool of rehabilitation professionals essentially including speech language pathologists (SLPs) at bachelors and masters (Rehabilitation Sciences) level and institutes. The role of these SLPs as health professionals is vital in identification, treatment and management of the paediatric and geriatric population, speech and language and communication disorders and feeding and swallowing disorders.^34^ The situation is better than in India where the brain drain is almost 48 % for SLP and hearing sciences post graduates.^35^

Sending patients home from acute medicine or emergency departments without rehabilitation causes them to suffer as a result they often end up dying from complications. Such a callous approach underscores the need to retain rehabilitation professionals and adopting these rehabilitative practices is an integral component of excellence in medical tourism to inspire confidence and trust in the system. In short, the brain drain has done more damage to health care systems in the developing countries than the benefit.^36^

The JCI accreditation for rehabilitation centres of academic learning and training and also clinical centres would boost national and international health tourism as well open opportunities for SLPs. Another significant advantage of international accreditation is that brain drain is considerably reduced as revenues start pouring into the medical and health sector which creates the fiscal space for procuring advanced medical instruments and machinery which will improve the quality of healthcare services. Tele health can be strengthened if international accreditation is achieved and the scope and extent of E health can be national or international. The patient or the “medical tourist” will make a beeline for a medical tourism hub if the language and culture is similar to his and he has beforehand checked and verified the credentials of the hospital where he intends to be treated. The health authorities in Pakistan have failed to make Pakistan attractive for medical tourism therefore the private hospitals in Pakistan and the medical and health professionals have to take up the cudgels to make Pakistan relevant in the international environment. Prior to seeking international accreditation research should be carried out to gauge the regional and national potential and ability of healthcare services to respond to an influx of visitors immediately after international accreditation is achieved. Crooks et al.^6^, have highlighted four issues catering to which may improve medical tourism prospects including material for promotion of such tourism which may also highlight advances in technologies. Developing states have to prove safe and current facilities and avoidance of claims of cost saving and low cost of treatment etc. may give a hidden message of poor quality. For Pakistan it is essential that government get involved to cater to the macroeconomic needs of medical tourism, facilitating foreigners as well as private sector including hospitals to meet the microeconomic needs including upgradation of hospitals.^37^

### Limitations:

Dearth of local literature on the subject was a limitation.

## CONCLUSION

It is dismal to note that the healthcare system in Pakistan is not geared towards national and international medical tourism compelling medical and healthcare professionals to move abroad. Patient care quality may not reach the desired level as few healthcare institutions possess international accreditation. To promote medical tourism private sector hospitals, health and medical and rehabilitation professionals will have to take the lead and awareness campaigns need to be aggressively launched. Initial capital investments would pay off in the medium term and the trickle of medical tourists can be substantially increased. To substantially increase medical tourism in Pakistan, legislation is not required and perhaps not even major executive decisions and the private sector can pull it off. Pakistani healthcare professionals may flock back once sufficient internationally accredited hospitals and centers are established providing medical tourists the comfort that international safety standards for patients are complied and quality care is available at comparable costs.
